# Quality of life before surgical ICU admission

**DOI:** 10.1186/1471-2482-7-23

**Published:** 2007-11-12

**Authors:** Fernando J Abelha, Cristina C Santos, Henrique Barros

**Affiliations:** 1 Department of Anesthesia and Intensive Care, Hospital de São João, Porto, Portugal.; 2 Biostatistics and Medical Informatics Department, Faculty of Medicine at the University of Porto, Portugal.; 3 Department of Hygiene and Epidemiology, University of Porto Medical Scholl, Porto, Portugal.

## Abstract

**Background:**

Examining the quality of life (QOL) of patients before ICU admission will allow outcome variables to be compared and analyzed in relation to it. The objective of this study was to analyze QOL of patients before admission to a surgical ICU and to study its relationship to outcome and to the baseline characteristics of the patients.

**Methods:**

All adult patients consecutively admitted to the surgical ICU between November 2004 and April 2005, who underwent non-cardiac surgery, were enrolled in this observational and prospective study. The following patient characteristics were recorded: age, gender, body mass index, ASA physical status, type and magnitude of surgical procedure, length of stay (LOS), in ICU and in hospital, mortality, Simplified Acute Physiology Score II (SAPS), history of co-morbidities and quality of life survey score (QOLSS). The relationships between QOLSS and ICU variables and outcome were evaluated. The relationship between the total QOLSS and each variable or outcome was assessed by multiple linear regression.

**Results:**

One hundred eighty seven patients completed the study. The preadmission QOLSS of the patients studied was 4.43 ± 4.90; 28% of patients had a normal quality of life (0 points), 38% had between 1 and 5 points (considered mild deterioration), 21% had between 6 and 10 points (moderate deterioration), 10% had between 11 and 15 points (considered major deterioration) and 3% had more than 15 points (severe limitation of quality of life). A worse preadmission QOLSS was associated with higher SAPS II scores, with older patients (age> 65 years) and with ASA physical status (ASA III/IV). Total QOLSS was significantly worse in elderly patients and in patients with co-morbidities and in patients more severely ill at ICU admission. Patients who died in the ICU and in hospital had worse QOLSS scores compared to those who survived. However, no statistical differences in QOLSS were found in relation to longer ICU stays (ICU LOS).

**Conclusion:**

Preadmission QOL correlates with age and severity of illness. Patients with co-morbidities and those who died during ICU or hospital stay had worse QOLSS scores.

## Background

The primary purpose of treatment in Intensive Care is to provide sufficient care to save lives and reduce morbidity in survivors. Improving functional capacity and quality of life should be the desired standards to achieve.

Although severity of illness has been found to be associated with greater length of stay in an ICU (LOS) and with ICU resource use [1-3], quality of life appears to be an important consideration for evaluating treatment benefits and resource allocation.

Studies of the quality of life in critically ill patients tend to focus on the results after discharge and very few have examined the situation before admission. Severity of illness and the presence of co-morbidities are parameters used to stratify patients on admission but the quality of life of these patients is seldom evaluated. The reasons are that quality of life has been considered an outcome not an evaluation parameter, and it is difficult to assess quality of life before admission. In a systematic literature review of quality of life in adult survivors of critical illnesses [4], only five studies [5-9] measured pre-admission QOL domains.

Rivera-Fernandèz et al. [10,11] validated an instrument to measure quality of life in critically ill patients at the moment of ICU admission. They verified that previous quality of life influenced quality of life after discharge and hospital mortality. This questionnaire fulfills all the criteria for application to critically ill patients on admission to an ICU [12] and has been used in other studies [13-16]. The study of quality of life before admission to ICU allows previous and subsequent situations to be compared with other variables measured on admission and during ICU stay that may be related to outcome.

The aim of the present study is to analyze QOL of patients before admission to a surgical ICU and to study its relationship to outcome and to the baseline characteristics of the patients.

## Methods

The study protocol was approved by our institutional review board and written consent was obtained from the patients or members of their families. The study was conducted in a surgical ICU that admits non-cardiac surgery patients for elective or emergency surgery. The criteria for ICU admission followed the guidelines of the Society for Critical Care Medicine[17]. All consecutive patients, admitted to the ICU between November 2004 and April 2005, were eligible for the study. Exclusion criteria were: age <18 years and death within the first 6 hours after admission. Patients readmitted during the study period were enrolled in relation to the time of their first admission.

The following clinical variables were recorded on admission to the ICU: age, sex, body weight and height, ASA physical status, emergency or scheduled surgery, magnitude of surgical procedure classified as major (surgery in which body cavities or major vessels are exposed to ambient temperature e.g. major abdominal, thoracic, vascular, or thoracic spine surgery with instrumentation, or hip arthroplasty), medium (surgery in which body cavities are exposed to a lesser degree e.g. appendectomy) or minor (superficial surgery). The ICU and in-hospital LOS and mortality were also recorded for all patients, and SAPS II was calculated using standard methods [18].

Any history of ischemic heart disease, congestive heart failure, cerebrovascular disease, hypertension, renal insufficiency, diabetes or hyperlipidemia was recorded. The presence of coexisting conditions was assessed using the secondary diagnoses of the International Classification of Diseases, Ninth Revision, Clinical Modification (ICD-9-CM). Diabetes mellitus was considered if previously diagnosed or if the patient received treatment with an oral hypoglycemic agent during hospitalization. Adapting a classification scheme developed by Lee and colleagues [19], we calculated a Revised Cardiac Risk Index (RCRI) score for each patient, assigning one point for each of the following risk factors: high-risk surgery, ischemic heart disease, cerebrovascular disease, renal insufficiency and diabetes mellitus. According to Lee, high-risk surgery includes all intrathoracic, intraperitoneal and suprainguinal vascular procedures.

Data about LOS in the hospital and hospital mortality were collected during the patient's stay in the hospital.

To assess QOL, a questionnaire specifically constructed and validated by Rivera-Fernandèz for critically ill patients was used [10]. It comprises 15 items grouped into three subscales: basic physiological activities (subscale 1, four items, score range 0–9 points), normal daily activities (subscale 2, eight items, score range 0–15 points), and emotional state (subscale 3, three items, score range 0–5 points). Subscale 1 explores oral communication, food intake and sphincter control; subscale 2 explores tolerance of effort, mobility, movements of precision, capacity for dressing, work or activities appropriate to age, and social relationships; and subscale 3 explores vitality, subjective well-being and feelings. The questionnaire was administered by trained physicians on admission or during the days following admission and was answered either by the patient or, if the patient was unable to respond, by a family member with full knowledge of the patient's situation. The questionnaire explores the baseline QOL situation during the 2 months before its administration. Each item is weighed and results are expressed as a profile or a score. A score of 0 points signifies normality and the score increases as QOL worsens, ranging from 0 (normality) to 29 (worst score) (additional file 1).

### Statistical methods

The data are expressed as mean ± sd. To compare two means we used Student's *t*-test. To compare more than two means we used analysis of variance (ANOVA). Multivariate analysis was performed by multiple linear regression, using QOLSS as the dependent variable and the "stepwise" method for selecting variables. The results are expressed as the coefficients of the equations and the values of the multiple regression coefficients at each step. The level of significance was 0.05 (two sided) for all statistical tests. SPSS for Windows version 13.0 (SPSS, Chicago, IL) was used to analyze the data.

## Results

One hundred and ninety patients were admitted to the ICU between November 2004 and April 2005. The QOL questionnaire was not completed for 3 patients because they could not express themselves and no close family member was available. The questionnaire was answered either by the patient (82%) or by a family member (18%). A total of 187 patients entered the study, and their demographic and clinical characteristics are presented in table 1. Sixty-seven percent were male, the mean (minimum-maximum) age was 65 years (23–88), mean SAPS II was 25.4 ± 15.2 (range 2–82) and mean LOS was 4.6 ± 8.1 days. Eleven (5.9%) patients died in the ICU and 4 (2.1%) patients died in the ward after ICU discharge.

**Table 1:** Demographics and clinical characteristics of admitted patients (n = 187)

**Variable**	**mean ± SD or median and range or number/total number**
Age	Median 65, mean 62.4 ± 14.2 (range, 23 – 88)
< 65/≥ 65	94/93
Male/Female (n/%)	125(66.8)/62 (33.2)
Body mass index (Kg/m2)	25.62 ± 6.0 (range, 12.4 – 54.4)
ASA Physical status	
I	17 (9.1)
II	66 (35.3)
III	86 (46.0)
IV	18 (9.6)
Emergency surgery	39 (21.0)
Magnitude of surgery	
Minor	27 (14.4)
Medium	77 (41.2)
Major	83 (44.4)
General anesthesia	155 (82.9)
Regional anesthesia	14 (7.5)
Combined anesthesia	18 (9.6)
Duration of anesthesia (min.)	258 ± 143 (range 60–840)
≤ 180 min.	45 (24.1)
> 180 min.	142 (75.9)
Hypertension	85 (45.5)
Hyperlipidemia	28 (15.0)
High-risk surgery	76 (40.6)
Ischemic heart disease	37 (19.8)
History of congestive heart disease	27 (14.4)
History of cerebrovascular disease	20 (10.7)
Insulin therapy for diabetes	11 (5.9)
Preoperative serum creatinine > 2.0 mg/dl	13 (7.0)
Revised cardiac risk index	
0	62 (33.2)
1	84 (44.9)
2	27 (14.4)
3	10 (5.3)
≥ 4	4 (2.1)
QOL total score	4.43 ± 4.90
Subscale I	0.35 ± 1.06
Subscale II	3.01 ± 3.52
Subscale III	1.08 ± 1.26
Score of Acute Physiologic system (SAPS II)	25.4 ± 15.2 (range 2–82)
Length of ICU stay (days)	4.6 ± 8.1 (range, 0.4 – 63)
< 7 days	159 (85)
≥ 7 days	28 [35]
Length of hospital stay (days)	median 18; percentile 25, 9; percentile 75, 33 range 1–211
Any major cardiac complication	
Ventricular fibrillation/cardiac ar rest	1 (0.5)
Acute Myocardial Infarction	9 (4.8)
Pulmonary edema	4 (2.1)
Mortality in the ICU	11 (5.90)
Mortality in the Hospital	15 (8.0)

ASA, American Society of Anesthesiologists; ICU, Intensive Care Unit; QOL, Quality of life

The preadmission QOLSS of the patients studied was 4.43 ± 4.90. Women had higher scores (4.69 ± 4.61) than men (4.31 ± 5.05) but the differences were not significant. Older patients (5.82 ± 5.40) had higher scores than younger patients (3.03 ± 3.89) and these differences were statistically significant. Table 2 shows the QOLSS values by age and gender.

**Table 2:** Mean ± SD and sample size for QOLSS at admission by gender and age (< 65 years and ≥ 65 years)

	**Gender**			
	**Female **(n = 62)		**Male **(n = 125)	
	<65 years (n = 26)	≥ 65 Years (n = 36)	<65 years (n = 68)	≥ 65 Years (n = 57)
**QOLSS**	2.56 ± 2.52	6.17 ± 5.15	3.22 ± 4.31	5.59 ± 5.59

QOLSS, Quality of life survey score

For subscale 1 the score was 0.35 ± 1.06, for subscale 2 the score was 3.01 ± 3.52 and for subscale 3 the score was 1.08 ± 1.26.

The percentage of people showing normality for each subscale was: 85% for subscale 1 (basic physiological activities), 71% for subscale 2 (normal daily activities) and 48% for subscale 3 (emotional state). For QOLSS, 52 patients (28%) had 0 points or normal quality of life, 38% had between 1 and 5 points (considered mild deterioration), 21% had between 6 and 10 points (considered moderate deterioration), 10% had between 11 and 15 points (considered major deterioration) and 3% had more than 15 points (considered severe limitation of quality of life).

Table 3 shows the mean ± SD for each QOLSS variable. Total QOLSS was significantly worse in elderly patients and in those with hypertension, cerebrovascular disease, renal insufficiency or who were more severely ill as measured by SAPS and ASA. Patients who died in the ICU and in hospital had worse QOLSS scores compared to those who survived. However, no statistical differences in QOLSS were found in relation to longer ICU stays (ICU LOS).

**Table 3:** Mean ± SD for QOLSS for each variable

**Variable**	**QOLSS mean ± SD**	**p**
Age (years)		**<0.001**
< 65 [35]	3.03 ± 3.89	
≥ 65 [35]	5.82 ± 5.40	
Gender		0.621
Female [35]	4.67 ± 4.60	
Male (125)	4.31 ± 5.05	
BMI		
BMI ≥ 30 (162)	4.13 ± 3.51	0.739
BMI< 30 [35]	4.48 ± 5.09	
ASA Physical status		**0.001**
I/II (83)	3.08 ± 3.93	
III/IV [35]	5.49 ± 5.31	
Type of surgery		0.104
Emergency surgery (39)	5.68 ± 6.63	
Routine surgery (148)	4.11 ± 4.31	
Magnitude of surgery		0.864
Minor/Medium [35]	4.38 ± 4.27	
Major (83)	4.51 ± 5.62	
Hypertension		**0.004**
Yes (81)	5,58 ± 5,44	
Not [35]	3.49 ± 4.19	
Hyperlipidemia		0.471
Yes [35]	4.55 ± 5.10	
No (159)	3.82 ± 3.65	
High-risk surgery		0.991
Yes (76)	4.43 ± 5.07	
No (111)	4.44 ± 4.80	
Ischemic heart disease		0.676
Yes (37)	4.13 ± 3.72	
No (150)	4.51 ± 5.17	
History of congestive heart disease		0.103
Yes [35]	5,85 ± 4.37	
No (160)	4,18 ± 4.96	
History of cerebrovascular disease		**0.011**
Yes (20)	4.11 ± 4.62	
No (167)	7.05 ± 6.26	
Insulin therapy for diabetes		0.990
Yes (11)	4.44 ± 4.98	
No (176)	4.46 ± 3.62	
Preoperative serum creatinine > 2 mg/dl		**0.012**
Yes (13)	4.18 ± 4.69	
No (174)	7.69 ± 6.38	
RCRI		
≥ 2 (41)	6.07 ± 5.08	**0.014**
< 2 (146)	3.95 ± 4.76	
SAPS II		**0.000**
<25 (102)	6.44 ± 3.94	
>25 (85)	3.12 ± 5.53	
Postoperative AMI		0.483
Yes [35]	5.56 ± 4.33	
No (179)	4.38 ± 4.93	
Length of ICU stay (days)*		0.094
< 7 days (157)	4.18 ± 4.67	
≥ 7 days [35]	5.89 ± 5.89	
Mortality in Hospital		0.016
No (172)	4.17 ± 4.84	
Yes [35]	7.33 ± 4.72	
Mortality in ICU		**0.047**
No (176)	4.25 ± 4.82	
Yes (11)	7.27 ± 5.50	

BMI, Body mass index; ASA, American Society of Anesthesiologists; RCRI, Revised cardiac risk index; SAPS II, Simplified Acute Physiology Score; AMI, Acute myocardial infarction; ICU, Intensive Care Unit

Table 4 shows the multivariate analysis with linear regression, estimating the coefficients of the linear equation with the independent variables involved (age, ASA, SAPS II, mortality in ICU and in hospital, RCRI risk factors age and history of hypertension) entered by the stepwise method. This analysis shows that worse preadmission QOLSS was associated with higher SAPS II scores, older patients (age> 65 years) and worse ASA physical status (ASA III/IV). Those variables were associated in the regression model when QOLSS was the dependent variable.

**Table 4:** Multivariate analysis by stepwise method between QOLSS and variables included in table 3 that had statistical significance.

**Variable**	**B**	**ST Error**	**p**
SAPS II	1.786	0.589	0.003
Age (>65 years)	1.839	0.702	0.010
ASA (III/IV)	1.743	0.700	0.014

ASA, American Society of Anesthesiologists; SAPS II, Simplified Acute Physiology Score. Adjusted for age, ASA, SAPS II, mortality in ICU and in hospital, RCRI risk factors r = 0.391 and r^2 ^= 0.153 for this model

## Discussion

In this study, 52% of patients had a normal quality of life before admission and only 13% had major to severe limitation. Statistically significant differences were observed in preadmission QOLSS with respect to age and severity of illness according to SAPS II scores and ASA physical status: a worse preadmission QOL was observed in older and more severely ill patients. Compared to the survivors, the patients who died in the ICU or in hospital had significantly higher QOLSS scores. These scores were significantly different in patients with co-morbidities such as hypertension, history of cerebrovascular disease and renal insufficiency. QOLSS was not significantly different in patients who stayed longer in the ICU.

As reported by others [11,16,20], the worsening of QOL with increasing age may reflect more limited physical faculties among elderly patients. The impact of age on QOL after ICU has been described by a number of investigators [8,21,22] with reference to worse QOL in older patients. However, the relationship between QOL and age is less clear than it might seem, since our results show that QOL is worse among older patients before ICU admission. This may indicate that QOLSS should be always adjusted for age.

Severity of illness scores could be seen as a determinant of QOL, as in other studies using APACHE III [6,11] or APACHE II [23], and may reflect the impact of acute physiological derangements on admission; so they are indirect indicators of co-morbidities (chronic disease accounts for severity scores) and age itself.

Patients with high severity of illness had significant co-morbidity and a poor baseline status, which seemed to be major contributors to the QOLSS [24]. In support of previous studies [14,25], QOL measurement is needed as a control for pre-existing impairment because it is difficult to determine whether QOL decrements at follow-up reflect the impact of critical illness or simply a lower baseline QOL. A possible explanation for this finding is that patients who already have an impaired QOL may have a reduced physiological (or psychological) reserve and be less able to tolerate the insult of serious illness and an ICU stay [20,26].

ASA physical status, a universal indicator in surgical patients, correlated independently with QOL. The ASA score, a preoperative evaluation used routinely for every patient, was never intended to be a perioperative risk score, but all large-scale studies have suggested that a high ASA score is one of the best predictors of postoperative morbidity [27,28]. This indicator itself depends on a more generic classification of the presence of disease, and in itself indicates the presence of co-morbidities.

As in other studies [11,29-31], we found no correlation between QOLSS and LOS, nor did patients who stayed longer in the ICU have significantly higher QOLSS values.

The patients who died in the ICU or in hospital had worse QOLSS on admission, confirming the importance of adequate QOL assessment on admission to allow a better study of the impact of critical illness and scheduled treatment. [11,32,33]. Also this was not our main objective these results are in agreement with the work of Hofhuis et al. that concluded that QOL before ICU admission can be used as a predictor of mortality [33].

Prior health status according to the patient's diagnosis confers different risks and probably a different ICU outcome. Therefore background variables, particularly age and previous health status, should always be taken into account when assessing outcome.

But, most of all our objectives were not to identify patients to whom surgery should be denied. Our aim was to understand outcome in surgical patients and to better understand how co morbidities or previous QOL influence post operative outcome. This should be considered important in planning post operative care to these patients and that can only be achieved if we understand the needs of these patients.

We used a validated quality of life questionnaire [10] to measure this complex and multidimensional concept that includes different facets such as functional capacity, physiological functions and subjective aspects. This questionnaire can be applied to all types of critical care patients and can be completed rapidly and easily by the patient or a close family member. It is valid as a follow-up study after discharge from the ICU but also during admission, allowing the patient's previous status to be better understood and current situations to be differentiated. A valuable feature of the survey is that it can be completed by a surrogate, given that many critical patients are sedated or in tracheal intubation and unable to respond. The questionnaire was specifically designed for use in the ICU, where patients are frequently unable to respond directly, and it has demonstrated high patient/family member reproducibility. Relatives' opinions about the quality of life of a critically ill patient have also been used to estimate the baseline QOL [34].

There are several limitations to our study including the potential bias inherent in the questionnaire and the conditions of its use. Only surgical patients were admitted and no diagnostic risk index was used to stratify their disease risk more precisely. Another limitation is that information would ideally be completed before admission to avoid bias in recall of health status, potentially overestimating the degree of baseline function. Pre-hospital information was not available with our study design. Although our sample size of 187 patients was adequate for the correlations performed, it was not sufficient to perform subset analyses of surgical ICU populations.

Admissions to ICU are not homogeneous, and generalizing these findings to all ICU admissions may be misleading since our results represent only a surgical population. The population studied mainly comprised patients undergoing scheduled surgery, probably already having a reduced QOL, and surgery was performed in an attempt to improve their QOL and survival. These patients are usually subjected to careful pre-surgery screening to determine the likelihood of success, and all these premises must be considered in analysing these results.

Our objective was not to identify patients to whom surgery should be denied or postponed and we did not pretend to use QOLSS as an indicator of final outcome. We wanted to understand how previous QOL influence post operative outcome. With this purpose the pre-admission QOLSS proved to be an important QOL assessment tool and it appears sensible enough to incorporate in clinical practice to be used for the assessment of HRQOL.

## Conclusion

In conclusion, measurement of QOL before ICU hospitalization in our study appears to correlate with severity of illness, co-morbidities and age, and consequently with outcome.

## Competing interests

The author(s) declare that they have no competing interests.

## Authors' contributions

FA participated in conception, design, acquisition of the data, analysis of the data, statistical analysis, critical revision of the manuscript and supervision.

CS participated in analysis of the data, statistical analyses and drafting of the manuscript.

HB has been involved in drafting the manuscript, analysis of the data and revising it critically for important content.

All authors read and approved the final manuscript.

## Pre-publication history

The pre-publication history for this paper can be accessed here:



## Supplementary Material

Additional file 1 appendix 1. Quality of life questionnaire. Click here for file
