# Supplementary figures and images for: Quality of life before surgical ICU admission
Source: BMC Surg. 2007 Nov 12;7:23. doi: 10.1186/1471-2482-7-23 (PMC2194661; doi:10.1186/1471-2482-7-23)

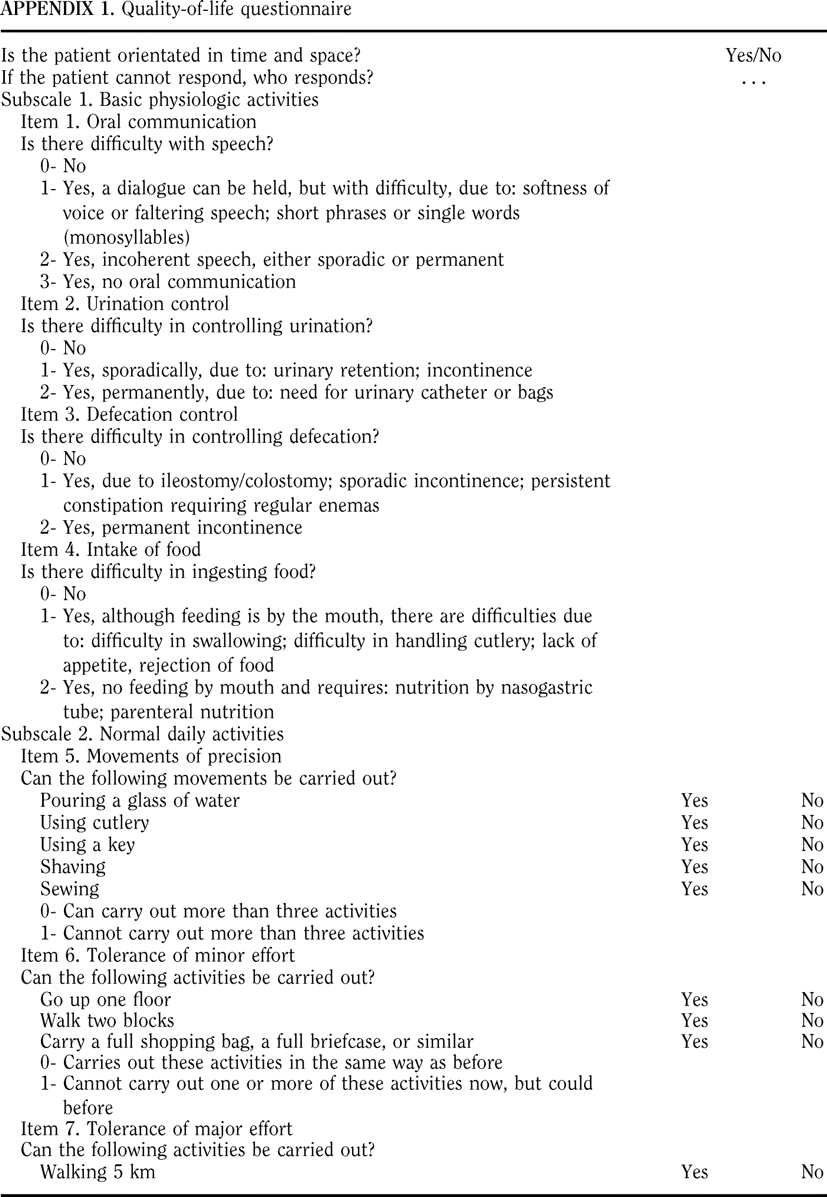


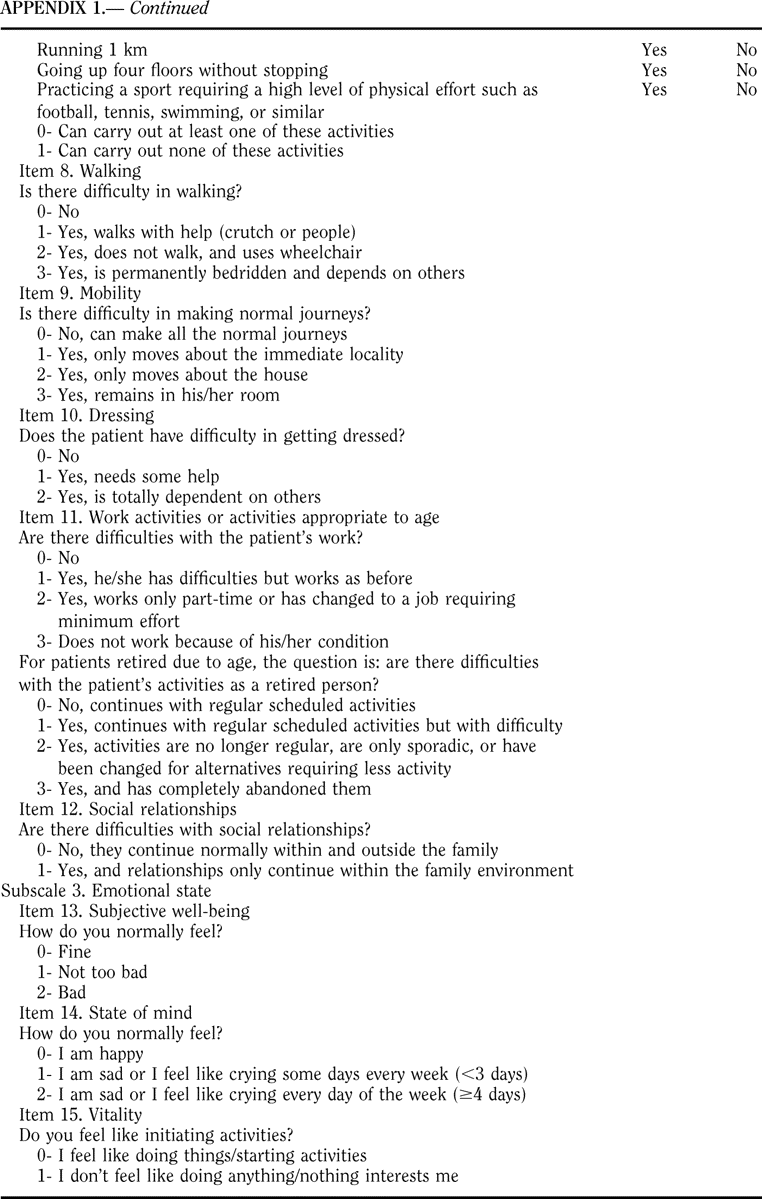

Supplement: Additional file 1 — appendix 1. Quality of life questionnaire. [file 1471-2482-7-23-S1.doc]
